# Evaluation of Cellular Responses by *Chlamydomonas reinhardtii* in Media Containing Dairy-Processing Residues Derived from Cheese as Nutrients by Analyzing Cell Growth Activity and Comprehensive Gene Transcription Levels

**DOI:** 10.3390/microorganisms12040715

**Published:** 2024-03-31

**Authors:** Akihito Nakanishi, Misaki Yomogita, Tomohito Horimoto

**Affiliations:** 1 School of Bioscience and Biotechnology, Tokyo University of Technology, Tokyo 192-0982, Japan; 2 Graduate School of Bionics, Tokyo University of Technology, Tokyo 192-0982, Japan; g112204255@edu.teu.ac.jp; 3 Meiji Co., Ltd., Tokyo 192-0919, Japan; tomohito.horimoto@meiji.com

**Keywords:** *Chlamydomonas reinhardtii*, whey, whey retentate, cellular response, transcriptomics, biomass, mineral, potassium

## Abstract

Utilities of whey powder (WP) and whey protein concentrate 34% powder (WPC34) prepared as dairy-processing residues were evaluated using a green alga *Chlamydomonas reinhardtii*. Analysis of *C. reinhardtii* growth showed that the strain used WP and WPC34 as nitrogen sources. Its specific growth rate and maximum cell density in WP-containing medium were higher than those in WPC34-containing medium; growth with WPC34 was improved by adding KCl or K_2_HPO_4_, which content was decreased as a result of WPC34’s preparation from WP. Although the lipid contents in media containing dairy-processing residues were 2.72 ± 0.31 wt% and 2.62 ± 0.20 wt% with no significant difference, the composition ratio of fatty acid C14 with WPC34 was higher than that with WP and the composition ratio of the sum of fatty acid-C16 and -C18 with WPC34 tended to be lower than that with WP. Additionally, analyses of gene transcription showed that the transcription level of *acetyl-CoA carboxylase biotin carboxyl carrier protein* in WPC34-containing medium was lower than that in WP-containing medium, possibly affecting the ratios of the chain lengths of fatty acids. The transcription of genes involved in glycolysis and the TCA cycle was outstandingly lower in algae grown in WPC34-containing medium when compared to those cultivated in the presence of WP, resulting in differences in energy production for cell proliferation.

## 1. Introduction

In the production of cheese using cow’s milk, approximately 90% of the raw cow’s milk yields whey as a byproduct [1]. Despite being a byproduct, whey is renowned for its nutritional value. The primary components of whey are water (comprising 93% of the total whey), lactose (C_12_H_22_O_11_, accounting for 70–72% of the total solids), whey proteins (8–10% of the total solids), and minerals (12–15% of the total solids). The minerals predominantly consist of calcium, potassium, sodium, and magnesium salts (half of which are the calcium salts NaCl and KCl), with trace amounts of metals such as zinc and copper [1]. Whey also contains minor constituents such as lactic acid, citric acid, non-protein nitrogen compounds (urea and uric acid), and vitamin B [2]. As shown above, whey contains high levels of nutrients so that the environmental impacts of whey usage should be considered in the dairy manufacturing industry [1,3]. The full utilization of whey has not been realized for two reasons: firstly, the membrane concentration process is an economies-of-scale process, thus small-scale cheese producers are unlikely to adopt it because they cannot achieve an efficient process; secondly, the issue of processing and utilizing cheese whey permeate, a by-product of whey protein concentrate production, remains unresolved. Therefore, research and technological development to solve these problems is needed, and an integrated approach to the social distribution of whey as a by-product has been studied under the concept of a circular economy [4,5]. A practical way to utilize whey as a by-product is as a high-quality protein source, and technology has been established to produce whey protein concentrate by concentrating the protein fraction from whey liquid [6,7]. This technology has been widely adopted in markets with large production scales, such as the United States, which accounts for approximately half of the world’s production and is the major supplier of whey [8]. On the other hand, we need to develop cheese whey application technologies that can be used in smaller markets.

Bioconversion through a microbial fermentation process is an example of a method to use cheese whey and its permeate [9,10,11,12,13,14]. Microorganisms called cell factories are able to carry out complex metabolic reactions continuously in a single cell [15]. For several decades now, *Saccharomyces cerevisiae* and *Escherichia coli* have been representative industrial microorganisms with merits such as safety [16] and capability of modification by genetic engineering [17], resulting in their use as bio-converters for value-added production [18,19]. In recent years, not only these heterotrophic microorganisms but also autotrophic microorganisms including green algae such as *Chlamydomonas reinhardtii* and *Chlorella vulgaris* have attracted attention for use as material producers [20,21,22]. These green algal species can be additionally useful as bio-converters because these species can convert inorganic carbon sources such as carbon dioxide and organic carbon sources like acetic acid into useful metabolites [23]. In particular, *C. reinhardtii* has been studied as a model species for a long time, starting with its use as a hydrogen-producing species in 1939. Many results obtained by researchers’ enthusiastic efforts have shown its safety [24], genetic modification [25,26], and 10–50 times higher CO_2_ fixation ability than general terrestrial plants [26]. Therefore, *C. reinhardtii* is a promising species of choice for research on constructing a carbon-cycling system in the food field to produce materials based on metabolic modification.

In this study, two dairy by-products, whey powder (WP) and whey protein concentrate 34% powder (WPC34), were prepared to explore the potential uses of whey. WP is a concentrated and spray-dried whey liquid, while WPC34 is a spray-dried retentate obtained after ultrafiltration of the whey liquid concentrate, showing a higher protein content of 34% in dry weight. From a microbiological perspective, the performance of WPC34 as a nutrient was evaluated by analyzing the responses of *C. reinhardtii* to broaden the understanding of whey effective use. Given that the responses of microbial cells could be influenced by substrates as the nutrient stimuli [27], the comparative cellular responses of *C. reinhardtii* to WPC34 and WP should be evaluated through transcriptome analyses. A transcriptional analysis is favored to assess cellular responses toward environmental conditions earlier than proteomic and/or metabolomic analyses [28]. In this study, the investigation was performed on a green alga *C. reinhardtii* to reveal three aspects as follows: its ability to use the dairy-processing residues as substrates; its trend to biosynthesize lipids as cell production using the dairy-processing residues; and its cellular response to adding the dairy-processing residues. These aspects are important to expand the use of dairy-processing residues, corresponding to an extension of whey use.

## 2. Materials and Methods

### 2.1. Culturing and Growth—Evaluation of Green Alga C. reinhardtii

*Chlamydomonas reinhardtii* strain C-9: NIES-2235 was purchased from the National Institute for Environmental Studies (Tsukuba-shi, Ibaraki, Japan). In this paper, *C. reinhardtii* strain used in this study mean *Chlamydomonas reinhardtii* strain C-9: NIES-2235.

For maintenance of the cells, *C. reinhardtii* was cultured in Modified Bold’s basal medium: 1.5 mM NaNO_3_, 0.22 mM K_2_HPO_4_, 0.30 mM MgSO_4_·7H_2_O, 0.17 mM CaCl_2_·2H_2_O, 0.43 mM KH_2_PO_4_, 0.43 mM NaCl, and other necessary components described in a previous report [29] in a photobioreactor as follows: volume: 100 mL in a glass vessel; light intensity: 150 µmol photons·m^−2^·s^−1^ with white fluorescent lamps; gas bubbling: 0.8% CO_2_ gas at 0.3 volume/volume/minute (vvm); temperature: 23 °C as room temperature.

For pre-cultivation and main cultivation of the cells, *C. reinhardtii* was cultured in modified TAP medium containing each dairy-processing residue as a nutrient. As dairy-processing residues, WP as cheese whey concentration and WPC34 with a protein content of more than 34 wt% of dry weight were provided by Meiji Co., Ltd. (Chuo-ku, Tokyo, Japan), and their nutrient compositions were surveyed by Japan Food Research Laboratories. Firstly, the modified TAP medium was defined as modified tris(hydroxymethyl)aminomethane-acetate phosphate medium without NH_4_Cl: 3.5 mM CaCl_2_·2H_2_O, 0.41 mM MgSO_4_·7H_2_O, 4.4 mM KH_2_PO_4_, 0.68 mM K_2_HPO_4_, 2.0 mM tris(hydroxymethyl)aminomethane, 17 mM acetic acid, and necessary components as described in a previous report [30]. Secondly, a series of WPC34- and WP-containing media was prepared, based on ammonium-depleted modified TAP medium. The final concentrations of dairy-processing residues were 1 g·L^−1^, 3 g·L^−1^, 6 g·L^−1^, and 10 g·L^−1^ for WPC34-containing media and 5 g·L^−1^, 10 g·L^−1^, 20 g·L^−1^, and 30 g·L^−1^ for WP-containing media. In each medium, *C. reinhardtii* was cultured as follows: volume: 100 mL in 300 mL-scaled Erlenmeyer flask; light intensity: 0 µmol photons·m^−2^·s^−1^; gas bubbling: none; temperature: 23 °C as room temperature; stirring rate: 200 rotations per a minute with a stirring bar.

The optical density (OD) of the broth was monitored at 700 nm wavelength with a spectrophotometer ASUV-1100 (AS ONE Corp., Osaka-shi, Osaka, Japan) to analyze the growth. The growth activity of *C. reinhardtii* in each medium condition was analyzed with relative growth rate (*µ*) and doubling time (*t_d_*), calculated by Equations (1) and (2). (*t*_1_: sampling time 1 (h); *t*_2_: sampling time 2 (h) (*t*_2_ > *t*_1_); *S*_1_: scale of concentration indicated by OD_700_ at *t*_1_; *S*_2_: scale of concentration indicated by OD_700_ at *t*_2_). The statistically analytic result was shown as the standard deviation (SD). The pH was measured with a LAQUA twin pH-11B (Horiba, Kyoto-shi, Kyoto, Japan).

(1) $$µ=\frac{ln\left(S_{2}/S_{1}\right)}{\left(t_{2}-t_{1}\right)}\left(h^{-1}\right)$$

(2) $$t_{d}=\frac{ln2}{{µ}_{max}}\left(h\right)$$

### 2.2. Evaluation of Composition and Concentration of Lipids in Cells with Gas Chromatography

Cells were broken with 0.5 mm glass beads, and thereafter the total extracted lipids were methyl esterified with a fatty acid methylation kit (Nacalai Tesque Inc., Kyoto-shi, Kyoto, Japan). The quality and quantity of fatty acid methyl esters were analyzed with a capillary gas chromatograph GC-2025 (Shimadzu Co., Kyoto-shi, Kyoto, Japan) equipped with a DB-23 capillary column (60 m, 0.25 mm internal diameter, 0.15 μm film thickness) (Agilent Technologies Inc., Santa Clara, CA, USA) followed by a previous method [31]. The statistically analytic result was shown as the SD. Heptadecanoic acid (Sigma Aldrich Co., St. Louis, MO, USA) was used as an internal standard, and rapeseed oil (Merck KGaA, Frankfurter Str., Darmstadt, Germany) was used as a quantitative standard.

### 2.3. Measurement of Levels of Gene Transcription

*C. reinhardtii* was cultivated in modified TAP with WPC34 adjusted to 6 g∙L^−1^ or WP adjusted to 20 g∙L^−1^ as mentioned above. First, approximately 5 mg of cells was collected by centrifugation at 21,500× *g* for 5 min. The collected cells were mixed with 50 µL of QIAzol Lysis Reagent (QIAGEN N.V., Chuo-ku, Tokyo, Japan) and shaken for 5 min. After keeping the samples at 23 °C for 5 min, 10 µL of chloroform was added and the samples were placed on ice for 3 min. The treated samples were centrifuged at 21,500× *g* for 15 min at 4 °C, then the supernatant was shaken with 25 µL of isopropanol and the mixture was placed at 23 °C for 10 min. The supernatant was discarded after centrifugation at 21,500× *g* for 10 min, and the precipitant was rinsed with 1 mL of 70% ethanol. The rinsed sample was dried with a lyophilizer Refrigerated CentriVap Benchtop Vacuum Concentrator (Labconco Corp., Kansas City, MO, USA) and the dried precipitant was dissolved in 10 µL of RNase-free water. The prepared sample as total RNA was used to synthesize complementary DNA (cDNA) using a ReverTra Ace qPCR RT Master Mix with a gDNA Remover (TOYOBO Co., Ltd., Osaka-shi, Osaka, Japan). With the cDNA, quantitative PCR (qPCR) was performed with THUNDERBIRD SYBR qPCR Mix (TOYOBO Co., Ltd.) using a StepOne Real-Time PCR System (Thermo Fisher Scientific Inc., Waltham, MA, US). The average threshold cycle values were evaluated throughout the logarithmic amplification phase and were normalized by the level of *ATPase beta chain of ATP synthase* (*ATPS*). The qPCR primers (Supplemental Table S1) were designed based on the Primer3Plus algorithm (https://dev.primer3plus.com/index.html, accessed on 7 April 2021), using information from each predicted gene sequence obtained via the genome information of NCBI. The statistically analytic result was shown as the SD.

## 3. Results

### 3.1. Compositions of Powders of WPC34 and WP

**Figure 1:**
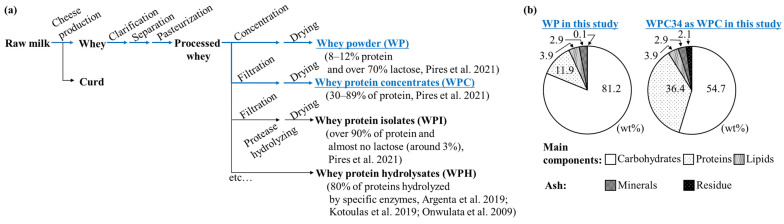
Preparatory processes of dairy residues and compositions of WPC34 and WP. WPC34 and WP as dairy-processing residue in this study. (**a**) Flow of preparatory process of WPC34 and WP from raw milk [32,33,34,35]; (**b**) Composition ratios of carbohydrates, proteins, and lipids as main components and minerals and residue as ash in WPC34 and WP.

In the series of several products including whey protein isolates [32] and whey protein hydrolysates [33,34,35] derived from whey, the composition characteristics of WPC34 and WP are presented in Figure 1b. WPC34 had 54.7 wt% carbohydrates, 36.4 wt% proteins, 3.9 wt% lipids, 2.9 wt% minerals, and 2.1 wt% residue (total 5.0 wt% as ash). On the other hand, WP had 81.2 wt% carbohydrates, 11.9 wt% proteins, 3.9 wt% lipids, 2.9 wt% minerals, and 0.1 wt% residue (total 5.1 wt% as ash). The filtration process decreased the carbohydrate ratio of WPC34 by a factor of 0.67 = 54.7/81.2 and increased the protein ratio by a factor of 3.1 = 36.4/11.9 compared to WP. There was little change in the composition ratio of total fat and ash.

### 3.2. Evaluation of Photo-Absorbances Derived from WPC34 and WP in Medium

**Figure 2:**
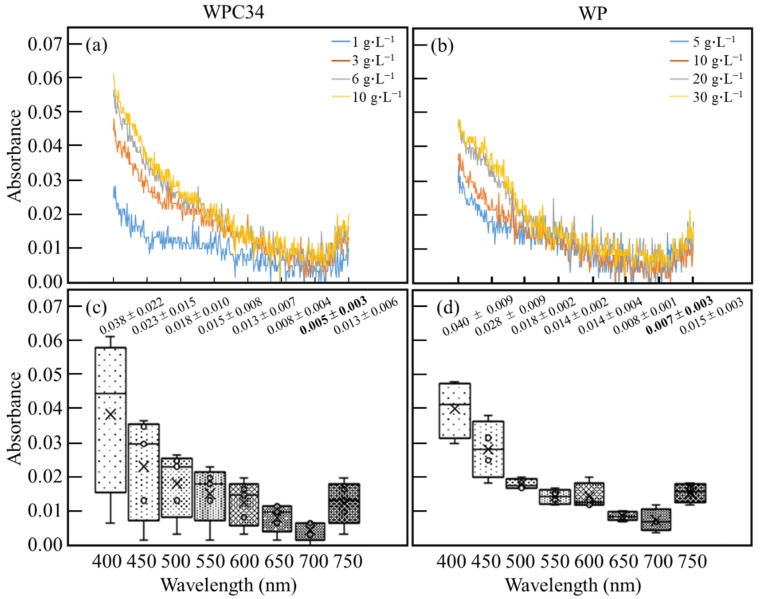
Analyses of spectra of photo-absorbances derived from WPC34 and WP in medium. Photo-absorbance spectra of media containing (**a**) 1 g∙L^−1^, 3 g∙L^−1^, 6 g∙L^−1^, and 10 g∙L^−1^ of WPC34 and (**b**) 5 g∙L^−1^, 10 g∙L^−1^, 20 g∙L^−1^, and 30 g∙L^−1^ of WP were analyzed in the 400–700 nm range. Box plots were drawn with absorbance data of media containing (**c**) 1 g∙L^−1^, 3 g∙L^−1^, 6 g∙L^−1^, and 10 g∙L^−1^ of WPC34 and (**d**) 5 g∙L^−1^, 10 g∙L^−1^, 20 g∙L^−1^, and 30 g∙L^−1^ of WP at each wavelength. The data used for the box plot analysis were three-time replicates for each concentration of dairy-processing residue (n = 3).

Absorbance characteristics derived from WPC34 and WP in the medium were optically analyzed with an absorbance spectrum from 400–750 nm (Figure 2a,b). The analyses revealed that the absorbances of both WPC34 and WP increased overall as WP concentrations increased, and the characteristics were particularly noticeable on the low wavelength side. Furthermore, the scattering of absorbances in each concentration of WPC34 and WP was also analyzed with boxplots (Figure 2c,d). In WPC34 (Figure 2c), the median value and the average value of absorbance separated toward the low wavelength side of 400 nm, and those values tended to coincide with each other at the high wavelength side of 700–750 nm. Furthermore, the scattering distribution of absorbance increased toward the low wavelength side of 400 nm and decreased at higher wavelengths such as 650–700 nm. In WP (Figure 2d), the values of the median and average absorbance from 400–750 nm tended not to be separated. In addition, the scattering distributions in the absorbance in the 500–750 nm range were lower than those in 400–450 nm. Furthermore, the absorbance of WPC34 and WP at 700 nm was 0.005 ± 0.003 and 0.007 ± 0.003, which were the lowest values in the absorption of each dairy-processing residue.

### 3.3. Evaluation of Growth of C. reinhardtii in TAP Medium Supplied with WPC34 and WP as Nutrients

**Figure 3:**
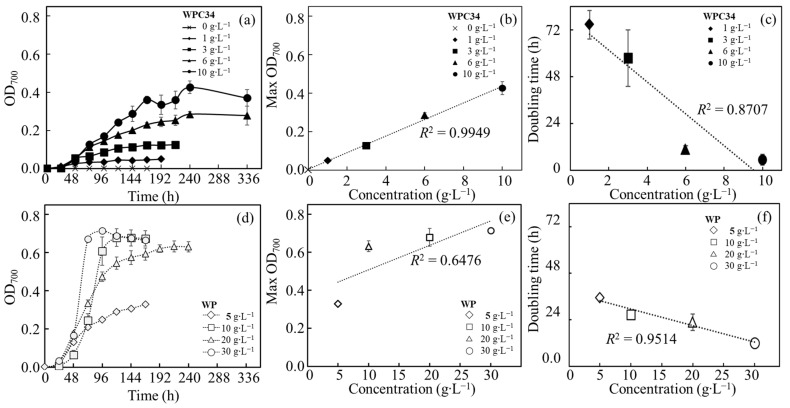
Analyses of time course profiling of OD_700_ related to *C. reinhardtii* growth in broth containing WPC34 and WP as nutrients. Assimilation activities of *C. reinhardtii* in media containing WPC34 and WP were analyzed with data obtained by 3–8 time-replicated experiments. Related to assimilating WPC34, (**a**) time course profiling of OD_700_, (**b**) maximum OD_700_ corresponding to WPC34 concentration, and (**c**) doubling time corresponding to WPC34 concentration are shown with closed symbols of diamond (1 g∙L^−1^), square (3 g∙L^−1^), triangle (6 g∙L^−1^), and circle (10 g∙L^−1^). Related to assimilating WP, (**d**) time course profiling of OD_700_, (**e**) maximum OD_700_ corresponding to WP concentration, and (**f**) doubling time corresponding to WP concentration are shown with open symbols of diamond (5 g∙L^−1^), square (10 g∙L^−1^), triangle (20 g∙L^−1^), and circle (30 g∙L^−1^), respectively. The data used for analysis were 3–9 time-replicates in each concentration of dairy-processing residue (n = 3–9), and error bars indicate the SD of 3–9 time-replicated experiments.

The time course profiling of OD_700_ was analyzed with *C. reinhardtii* cultured in modified TAP medium containing WPC34 or WP (Figure 3, Table 1). No increment of OD_700_ was observed in modified TAP medium without WPC34 or WP. In the WPC34-containing media, increments of the values of maximum OD_700_ were monitored as 0.05 ± 0.00, 0.12 ± 0.00, 0.28 ± 0.01, and 0.43 ± 0.03 in the medium containing 1 g∙L^−1^, 3 g∙L^−1^, 6 g∙L^−1^, and 10 g∙L^−1^ of WPC34, respectively. The correlation between the values of maximum OD_700_ and the values of WPC34 concentrations in each medium was analyzed, and the result showed a significant coefficient of determination (*R*^2^ = 0.9949). The maximum specific growth rates were obtained approximately in 24–96 h, and the doubling times calculated from those rates were shortened and this effect depended on the increment of WPC34 concentrations. Based on these results, the correlation was supported with a high coefficient of determination (*R*^2^ = 0.8707). In WP-containing media, increments showed as 0.33 ± 0.00, 0.63 ± 0.03, 0.69 ± 0.05, and 0.71 ± 0.01 in the media containing WP prepared as 5 g∙L^−1^, 10 g∙L^−1^, 20 g∙L^−1^, and 30 g∙L^−1^ of WP, respectively. The correlation between the maximum value of OD_700_ and the WP concentration in the medium was investigated, resulting in *R*^2^ = 0.6476. The maximum specific growth rates were obtained approximately in 48–96 h, showing that the doubling time calculated from those rates was shortened depending on the increment of WP concentrations. These data were analyzed, and the results reinforced the correlation between the doubling time and the WP concentration with a high coefficient of determination (*R*^2^ = 0.9514). In TAP medium, the *C. reinhardtii* used in this study displayed 0.461 ± 0.01 as the maximum OD_700_, 0.036 ± 0.004 as *µ*_max_ in 0–24 h, and 19.22 ± 2.10 as the doubling time, respectively.

The abundance ratio of each mineral in WPC34 and WP was quantified (Supplemental Table S1). For both WPC34 and WP, the major mineral was K at 11.2 mg∙g^−1^ and 13.4 mg∙g^−1^. The ratios of other minerals such as Ca, P, Na, and Mg showed contents of mg∙g^−1^, while those of Fe, Zn, Mo, and Cu indicated lower contents of µg∙g^−1^. The proteins were concentrated in WPC34 obtained from WP via filtration. WPC34 and WP used in this study contained proteins at 36.4 wt% and 11.9 wt% (Figure 1b), respectively, meaning that the concentration rate was approximately 3.1 times. Therefore, the mineral maintenance ratio was determined using the calculated values constructed of the 3.1-multiplied mineral concentration in WP as the denominator and the mineral concentration in WPC34 as the numerator. As a result of these analyses, the maintenance ratios of the major mineral components K, Ca, P, Na, and Mg were less than 50%; the ratios of Fe, Zn, Mo, and Cu, which had lower contents than the major mineral components, were more than 50%; especially for Fe, Zn, and Cu, the ratios exceeded 90%.

**Figure 4:**
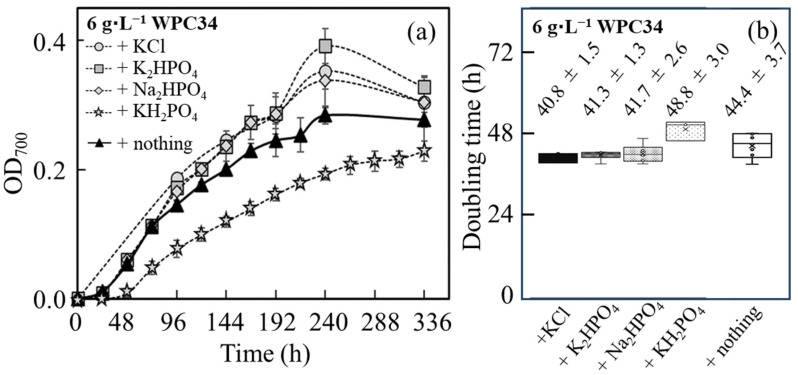
Analysis of time course profiling of OD_700_ related to *C. reinhardtii* growth in WPC34 broth containing minerals as additional nutrients. *C. reinhardtii* grown in 6 g∙L^−1^ WPC34-containing media with KCl (5.1 mM), K_2_HPO_4_ (2.6 mM), Na_2_HPO_4_ (2.2 mM), and KH_2_PO_4_ (2.6 mM) as additional minerals. (**a**) Time course profiling of OD_700_ is drawn with symbols of gray circle (KCl), gray square (K_2_HPO_4_), gray diamond (Na_2_HPO_4_), gray star (KH_2_PO_4_), and closed triangle (nothing). (**b**) Doubling times of OD_700_ in 6 g∙L^−1^ WPC34-containing media with KCl, K_2_HPO_4_, Na_2_HPO_4_, and KH_2_PO_4_ in 0–96 h are displayed from left to right, respectively. The data were obtained by 3–6 time-replicate experiments, and the error bars indicate the SD of 3–6 repeats.

The time course profiling of growth of *C. reinhardtii* shown as the OD_700_ was analyzed in WPC34-containing medium supplemented with K, P, and Na, which had low maintenance ratios in WPC34 (Figure 4a). WPC34 was produced by concentrating whey with a filter, and its concentration ratio is approximately shown by its protein content. The concentration ratio in this study was 3.1 times. Few nutrients were left out by filtration, meaning that 6–7 g of WPC34 was equal to 20 g of WP. Therefore, the concentrations of 20 g·L^−1^ of WP and 6 g·L^−1^ of WPC34 were mainly used in this study. 

Under all culture conditions, the maximum values of OD_700_ were reached in 240 h, resulting in the values of 0.352 ± 0.028, 0.391 ± 0.027, 0.338 ± 0.043, and 0.193 ± 0.009 with the addition of KCl, K_2_HPO_4_, Na_2_HPO_4_, and KH_2_PO_4_, respectively, and that was 0.284 ± 0.014 with no mineral-addition. Furthermore, compared to the condition without mineral addition, the maximum OD_700_ significantly increased under the conditions with KCl and K_2_HPO_4_ addition and tended to increase in the cultures enriched with Na_2_HPO_4_. On the other hand, the maximum OD_700_ was significantly decreased in the series with KH_2_PO_4_. Furthermore, regarding the values of OD_700_ affected by cell growth, the doubling time was analyzed during 96 h at the initial stage of the culture (Figure 4b). As a result, there was no significant difference in doubling time under all mineral-added conditions compared to the control. However, compared to the series without extra mineral addition, the doubling time under the conditions with the addition of KCl, K_2_HPO_4_, and Na_2_HPO_4_ tended to be shorter, and the doubling time under the condition with the addition of KH_2_PO_4_ tended to increase. For the aspect of the pH, the values of pH during 96 h were within 7–8 for all culture conditions tested.

### 3.4. Contents and Compositions of Lipid in C. reinhardtii Cells in Media Containing WPC34 and WP

**Figure 5:**
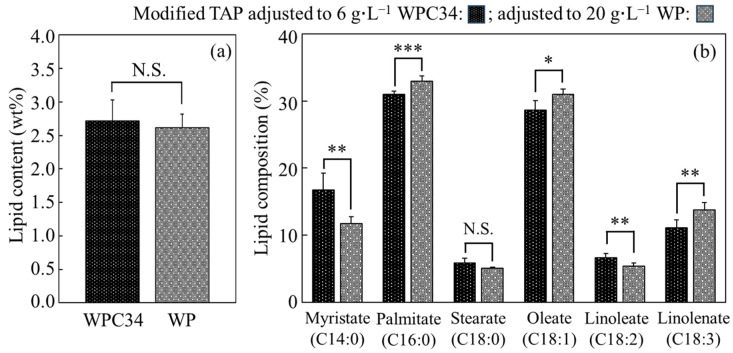
Lipid contents and compositions in *C. reinhardtii* cells cultivated in each broth. Lipids of *C. reinhardtii* cells cultivated in modified TAP adjusted to 6 g∙L^−1^ WPC34 and 20 g∙L^−1^ WP for 72 h were measured in terms of contents (**a**) and composition (**b**). The data obtained by 6–8 time-replicate experiments display error bars indicating the SD, and they were evaluated with significance tests (*: *p* < 0.05; **: *p* < 0.01; ***: *p* < 0.001; N.S.: not significant).

The contents and composition of lipids in cells cultured in modified TAP medium containing WPC34 and WP were analyzed (Figure 5). The lipid contents of the cells cultured with WPC34 and WP for 72 h were 2.72 ± 0.31 wt% and 2.62 ± 0.20 wt%, respectively, resulting in no significant difference. On the other hand, the lipid composition showed significant differences in several respects. In detail, firstly, 16.75 ± 2.48 wt% of the composition ratio of myristic acid as a fatty acid C14 in the medium containing WPC34 was significantly higher than 11.78 ± 1.00 wt% of that in the medium containing WP (** *p* < 0.01). Secondly, on the other hand, 30.97 ± 0.50 wt% of the composition ratio of palmitic acid as a fatty acid C16 in the medium containing WPC34 was significantly lower than 32.96 ± 0.80 wt% of that in the medium containing WP (*** *p* < 0.001); 52.28 ± 3.89 wt% as the total composition ratio of fatty acid-C18 in the cells with WPC34 was also significantly lower than the 55.26 ± 2.53 wt% observed for WP (* *p* < 0.05).

### 3.5. Evaluation of Cell Responses of C. reinhardtii by Analyzing Gene Transcription Levels in Each Broth Containing WPC34 and WP

**Figure 6:**
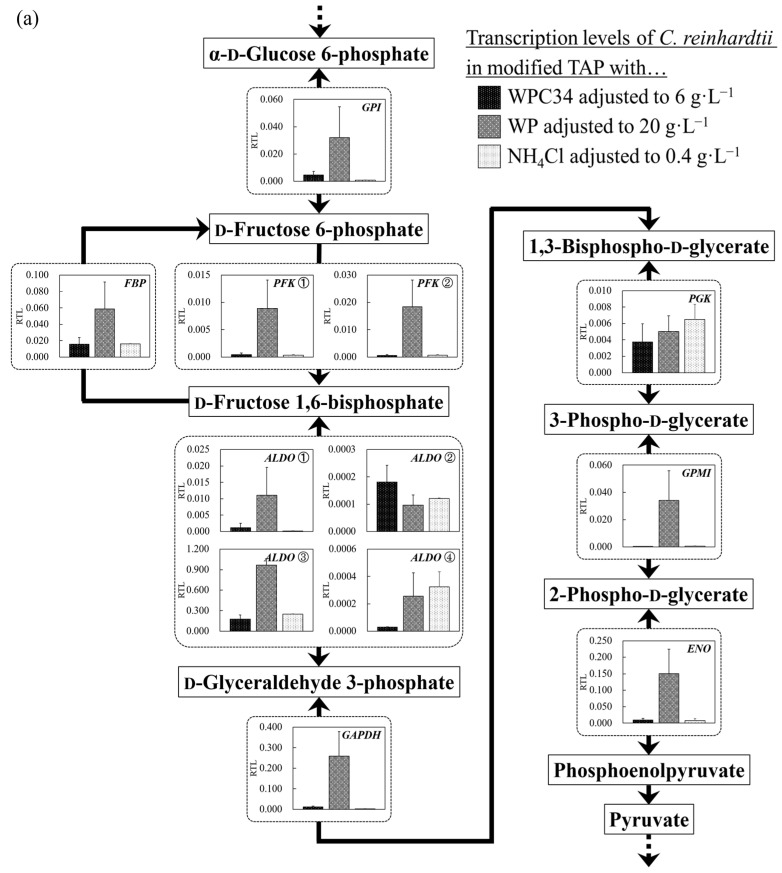

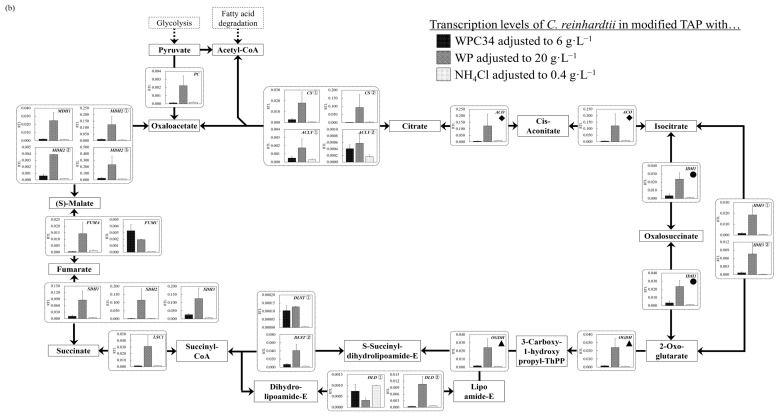

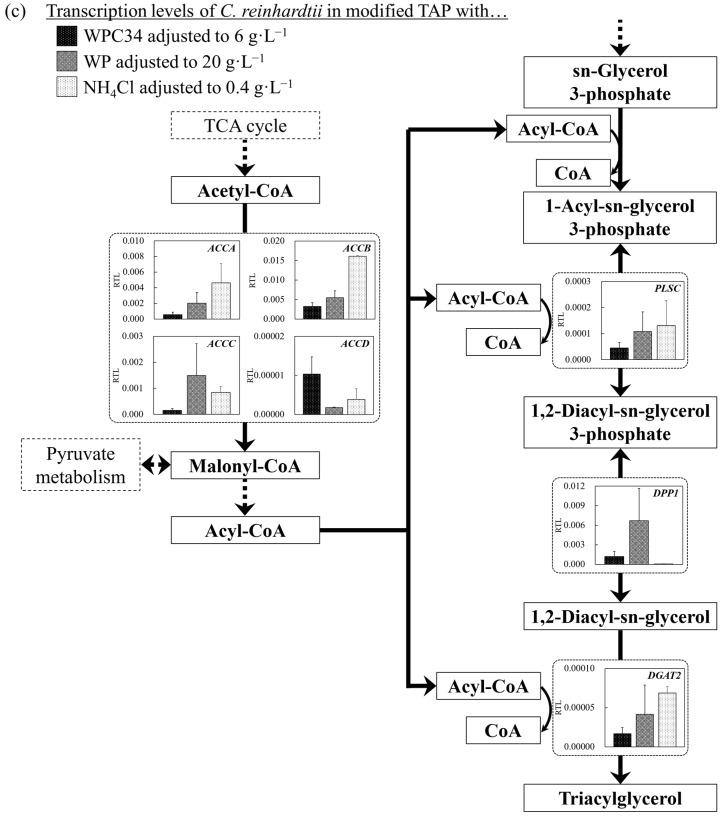

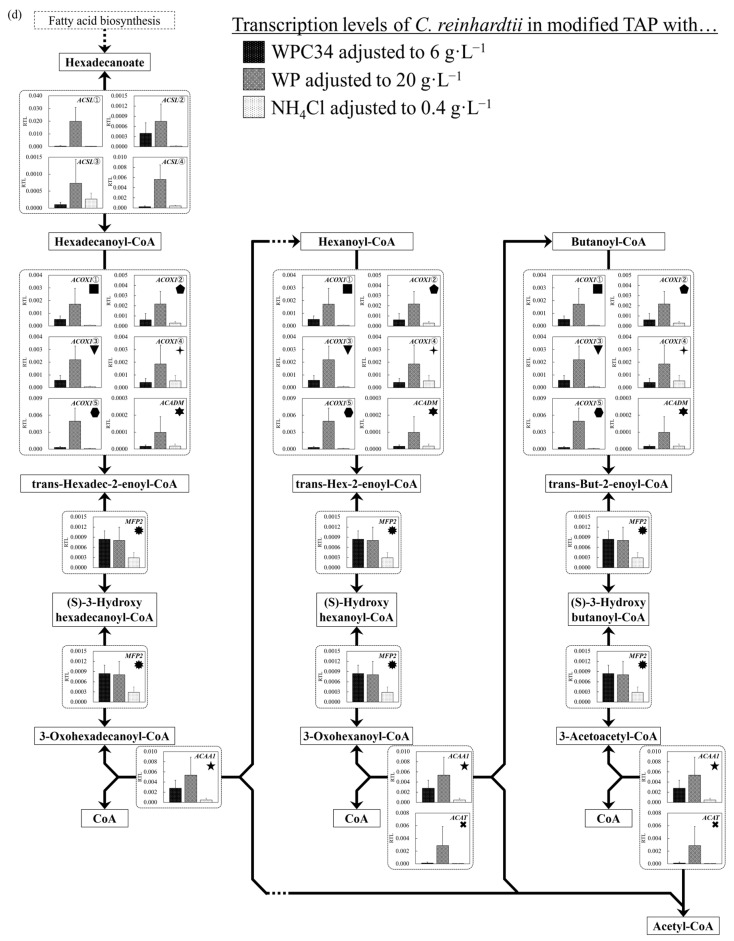
(**a**–**d**) Comparison of transcription levels of the genes whose products participate in glycolysis, TCA cycle, fatty acid synthetic pathway, and fatty acid degradation pathway of *C. reinhardtii* cells in each broth. Data are shown as relative mRNA transcription levels normalized by the level of *ATPS* as a housekeeping gene. The transcription levels at growth phase are displayed with a black bar (6 g∙L^−1^ WPC34), shaded bar (20 g∙L^−1^ WP), and gray bar (0.4 g∙L^−1^ NH_4_Cl) in the figure. The explanation of relative transcription level was abbreviated as RTL. Error bars indicate SD of 3–8 time-replicate experiments. Same shapes (such as square, triangle etc…) in figure meant the same genes. Gene abbreviations are shown below: (**a**) **in glycolysis.** *GPI*: *glucose-6-phosphate isomerase*, *FBP*: *fructose-1,6-bisphosphatase I*, *PFK*①: *6-phosphofructokinase 1*, *PFK*②: *6-phosphofructokinase* 1, *ALDO*①: *fructose-bisphosphate aldolase, class I*, *ALDO*②: *fructose-bisphosphate aldolase, class I*, *ALDO*③: *fructose-bisphosphate aldolase, class I*, *ALDO*④: *fructose-bisphosphate aldolase, class I*, *GAPDH*: *glyceraldehyde 3-phosphate dehydrogenase (phosphorylating)*, *PGK*: *phosphoglycerate kinase*, *GPMI*: *2,3-bisphosphoglycerate-independent phosphoglycerate mutase*, *ENO*: *enolase*. (**b**) **in TCA cycle.** *PC*: *pyruvate carboxylase*, *CS*①: *citrate synthase*, *CS*②: *citrate synthase*, *ACLY*①: *ATP citrate (pro-S)-lyase*, *ACLY*②: *ATP citrate (pro-S)-lyase*, *ACO*: *aconitate hydratase*, *IDH1*: *isocitrate dehydrogenase*, *IDH3*①: *isocitrate dehydrogenase (NAD+)*, *IDH3*②: *isocitrate dehydrogenase (NAD+)*, *OGDH*: *2-oxoglutarate dehydrogenase E1 component*, *DLD*①: *dihydrolipoyl dehydrogenase*, *DLD*②: *dihydrolipoyl dehydrogenase*, *DLST*①: *2-oxoglutarate dehydrogenase E2 component (dihydrolipoamide succinyltransferase)*, *DLST*②: *2-oxoglutarate dehydrogenase E2 component (dihydrolipoamide succinyltransferase)*, *LSC1*: *succinyl-CoA synthetase alpha subunit*, *SDH1*: *succinate dehydrogenase (ubiquinone) flavoprotein subunit*, *SDH*2: *succinate dehydrogenase (ubiquinone) iron-sulfur subunit*, *SDH3*: *succinate dehydrogenase (ubiquinone) cytochrome b_560_ subunit*, *FUMA*: *fumarate hydratase, class I*, *FUMC*: *fumarate hydratase, class II*, *MDH1*: *malate dehydrogenase*, *MDH2*①: *malate dehydrogenase*, *MDH2*②: *malate dehydrogenase*, *MDH2*③: *malate dehydrogenase*. (**c**) **in fatty acid synthetic pathway.** *ACCA*: *acetyl-CoA carboxylase carboxyl transferase subunit alpha*, *ACCB*: *acetyl-CoA carboxylase biotin carboxyl carrier protein*, *ACCC*: *acetyl-CoA carboxylase, biotin carboxylase subunit*, *ACCD*: *acetyl-CoA carboxylase carboxyl transferase subunit beta*, *PLSC*: *1-acyl-sn-glycerol-3-phosphate acyltransferase*, *DPP1*: *diacylglycerol diphosphate phosphatase/phosphatidate phosphatase*, *DGAT2*: *diacylglycerol O-acyltransferase 2*. (**d**) **in fatty acid degradation pathway.** *ACSL*①: *long-chain acyl-CoA synthetase*, *ACSL*②: *long-chain acyl-CoA synthetase*, *ACSL*③: *long-chain acyl-CoA synthetase*, *ACSL*④: *long-chain acyl-CoA synthetase*, *ACOX1*①: *acyl-CoA oxidase*, *ACOX1*②: *acyl-CoA oxidase*, *ACOX1*③: *acyl-CoA oxidase*, *ACOX1*④: *acyl-CoA oxidase*, *ACOX1*⑤: *acyl-CoA oxidase*, *ACADM*: *acyl-CoA dehydrogenase*, *MFP2*: *enoyl-CoA hydratase/3-hydroxyacyl-CoA dehydrogenase*, *ACAA1*: *acetyl-CoA acyltransferase 1*, *ACAT*: *acetyl-CoA C-acetyltransferase*.

For evaluation of the gene transcription levels by quantitative PCR, the scattering in the amounts of transcription of the reference gene (housekeeping gene) should be small under each culture condition. In this study, the comprehensive gene transcription levels of *C. reinhardtii* cultured in WPC34-containing medium, WP-containing medium, and regular TAP medium during logarithmic growth were investigated (Figure 6) so that the scattering of the reference genes could be analyzed in each medium. As the reference genes, *ATPS* [36], *actin-related protein Arp2/3 complex* [37], *subunit ARP2* (*Arp2*) [38], *actin-related protein Arp2/3 complex*, *subunit Arp3 gene* (*Arp3*) [38], *actin-related protein Arp2/3 complex,* and *subunit ARPC4 gene* (*Arpc4*) [38] were picked up for qPCR analysis using cDNA prepared from the strain in each medium (Supplemental Figure S1). *ATPS* turned out to be the most stable reference gene because of low scattering; therefore, it was chosen for further analyses. All information concerning reference genes and structural genes was obtained from the Kyoto Encyclopedia of Genes and Genomes and the National Center for Biotechnology Information.

The transcription levels of the genes encoding enzymes involved in glycolysis were analyzed. The levels in the WPC34-containing medium tended to be significantly lower than those occurring in the WP-containing medium and to be almost the same as those in the TAP medium. Especially, the transcription level of glyceraldehyde 3-phosphate dehydrogenase (GAPDH), an enzyme that acts in the border between the preparatory phase and pay-off phase, in the WP-containing medium was significantly higher than that in the WPC34-containing medium (** *p* < 0.01). The transcription levels of fructose-1,6-bisphosphatase I (FBP) and 6-phosphofructokinase 1 (PFK① and PFK②), enzymes that are indicators to switch between assimilation and dissimilation, and those of unique genes like 2,3-bisphosphoglycerate-independent phosphoglycerate mutase (GPMI) and enolase (ENO) controlling the metabolism from 3-phospho-d-glycerate to phosphoenolpyruvate in the WP-containing medium were significantly higher than those in the WPC34-containing medium (* *p* < 0.05, * *p* < 0.05, ** *p* < 0.01, * *p* < 0.05, ** *p* < 0.01) and in TAP medium (* *p* < 0.05, * *p* < 0.05, ** *p* < 0.01, * *p* < 0.05, ** *p* < 0.01). On the other hand, the transcription level of phosphoglycerate kinase (PGK) controlling the metabolic reaction between 1,3-bisphospho-d-glycerate and 3-phospho-d-glycerate showed no significant difference among all conditions.

The transcription levels in the TCA cycle were also analyzed (Figure 6b). On the whole, the gene transcription levels observed for the algae grown in the WPC34-containing medium tended to be lower than those observed for the WP-containing medium, and showed few differences from those in TAP media. Especially, the unique genes of pyruvate carboxylase (PC), aconitate hydratase (ACO), isocitrate dehydrogenase (IDH1), 2-oxoglutarate dehydrogenase E1 component (OGDH), and succinyl-CoA synthetase alpha subunit (LSC1) in the WPC34-containing medium were significantly lower than those in the WP-containing medium (* *p* < 0.05, * *p* < 0.05, ** *p* < 0.01, ** *p* < 0.01, ** *p* < 0.01). Although *isocitrate dehydrogenase* (*NAD^+^*) (*IDH3*① *IDH3*②), *succinate dehydrogenase* (*ubiquinone*) *flavoprotein subunit* (*SDH*1) *succinate dehydrogenase* (*ubiquinone*) *iron-sulfur subunit* (*SDH2*) *succinate dehydrogenase* (*ubiquinone*) *cytochrome b_560_ subunit* (*SDH3*) and *malate dehydrogenase* (*MDH1 MDH2*① *MDH2*② *MDH2*③) were not unique, those genes also showed a similar trend to the unique genes *PC*, *ACO*, *IDH1*, *OGDH,* and *LSC1*. In addition, compared to the transcription level of 2-*oxoglutarate dehydrogenase E2 component* (*dihydrolipoamide succinyltransferase*) (*DLST*①, *DLST*②) in TAP medium, they were significantly higher in the WPC34-containing medium (*** *p* < 0.001, ** *p* < 0.01) and in the WP-containing medium (*** *p* < 0.001, ** *p* < 0.01). Furthermore, compared to the transcription level of *dihydrolipoyl dehydrogenase* 2 (*DLD*②) in TAP medium, that in WPC34 was similar, while that in WP was significantly increased.

The transcription levels in lipid-producing pathways were investigated (Figure 6c). Most of the gene transcription levels in the WP-including medium did not show outstanding values like in the glycolysis pathway and TCA cycle, excluding that of *diacylglycerol diphosphate phosphatase/phosphatidate phosphatase* (*DPP1*). Regarding the reaction from acetyl-CoA to malonyl-CoA, the gene transcription levels of *acetyl-CoA carboxylase biotin carboxyl carrier protein* (*ACCB*) and *acetyl-CoA carboxylase carboxyl transferase subunit beta* (*ACCD*) in the WPC34-containing medium were significantly lower and higher than those in the WP-containing medium, respectively (* *p* < 0.05; * *p*< 0.05). In the pathway synthesizing lipids with acyl-CoA based on sn-glycerol 3-phosphate, the gene transcription levels of 1*-acyl-sn-glycerol-3-phosphate acyltransferase* (*PLSC*) and *diacylglycerol O-acyltransferase* 2 (*DGAT*2) in the WPC34-containing medium tended to be lower than those in the WP-containing medium and TAP medium.

Next, the gene transcription levels in lipid degradation pathways were analyzed (Figure 6d). The levels of *enoyl-CoA hydratase/3-hydroxyacyl-CoA dehydrogenase* (*MFP2*) and *acetyl-CoA acyltransferase 1* (*ACAA1*) in the dairy-processing residue-containing media were higher than those in the TAP medium, and especially those in the WPC34-containing medium showed a significant difference when compared to results obtained for TAP medium (* *p* < 0.05). The transcription levels of *long-chain acyl-CoA synthetase* (*ACSL*①, *ACSL*②, *ACSL*③ and *ACSL*④) in the WPC34-containing medium tended to be lower than those in the WP-containing medium, and the levels of *ACSL*① and *ACSL*③ especially displayed significant differences (* *p* < 0.05; ** *p* < 0.01).

Additionally, the levels of *acyl-CoA oxidase* (*ACOX1*①–⑤) and *acyl-CoA dehydrogenase* (*ACADM*) in the dairy-processing residue-containing media (especially in the WP-containing medium) tended to be higher than those in TAP medium.

## 4. Discussion

The composition of WPC34 and WP were analyzed in order to evaluate the use of WPC34 obtained as a retentate from whey by filtration (Figure 1). As a premise of the evaluation, the composition ratio of WPC34 and WP was the relative ratio of each composition; the absolute amounts of the constituent substrates in WPC34 were lower than those in WP by filtration; the ratios of the components were increased for components that were more likely to be retained in the filtration process and decreased for components that were less likely to be retained. The proteins in the whey are normally not discharged by the filtration but remain as retentate, and the amounts of proteins in WPC34 in this study could also be retained similar to those of general WP and WPC. 

The carbohydrates difficultly remained in the residue rather than proteins. So far, researchers have reported that the permeate obtained from whey through filtration contains a large amount of lactose [39], and thus it is known that lactose is reduced in the retentate. Therefore, the decrease in carbohydrate content in WPC34 could mean that lactose mainly decreased due to the filtration. 

In detail, ultrafiltration with a cutoff of 10 kDa or greater is an established process for producing WPC from WP [40]. This process concentrates the major whey proteins, such as β-lactoglobulin, α-lactalbumin, immunoglobulins, and serum albumin. However, UF allows lactose and minerals to permeate the membrane and be removed, resulting in a higher protein concentration [41]. The lipids in cheese whey and milk are known to be distributed as milk fat globules with a diameter of 100 to 15,000 nm [42]. Since whey proteins are 3 to 6 nm in diameter [42], milk fat globules have a larger molecular size than these. This implies that lipids should be enriched to the same extent as proteins in WPC with UF treatment of whey concentrate. However, the fact that they were comparable in WP and WPC34 suggests that lipids may have been removed in some way during the other manufacturing steps of processing the whey concentrate into WPC. Regarding the minerals, processing cheese whey or milk by UF reduces the concentration of monovalent cations such as sodium and potassium by permeation, but divalent cations such as calcium are retained and concentrated [43]. Since the changes in sodium, potassium, and calcium values in Supplemental Table S2 showed the same changes as those described above, this means that the concentration or removal of various minerals does not significantly affect the ash values.

*Chlamydomonas reinhardtii* strain C-9: NIES-2235 used in this study is a mating type (-), and vegetative cells, which are haploid zoospores, grow through an asexual reproduction cycle. The species has a state in which it encapsulates multiple cells during the growth process in the asexual reproduction cycle, and it is difficult to evaluate growth simply by cell counting, as in the case of the budding yeast *Saccharomyces cerevisiae*. Additionally, on a laboratory scale, a small amount of *C. reinhardtii* cells is difficult to collect and weigh with accuracy so it is also difficult to evaluate growth simply by cell weight. The fact that not only the cell density but also the cell size influenced the OD value should be considered because each cell becomes large in the asexual reproduction phase when a cell contains multiple cells. This condition is a part of the total life cycle so the growth of *C. reinhardtii* has been evaluated based on the OD value [44]. 

As the results of the spectrum analyses for the dairy-processing residues to evaluate whether dairy-processing residues influence the wavelength are deeply related to the cell growth (Figure 2), the absorbance of both WPC34 and WP increased and this depended on the increased concentrations of those residues in the low wavelength side around 400 nm (Figure 2a,b), meaning that the absorbances here were derived from those residues. In WPC34 conditions (Figure 2c), the values of the median and average of absorbance separated in the lower wavelength band around 400 nm, and these values tended not to separate from each other at higher wavelengths such as 700–750 nm, meaning that the concentrations of WPC34 in the lower wavelength range could affect the absorbance and those in the higher wavelength range were difficult to influence. On the other hand, in WP conditions (Figure 2d), the values of the median and average absorbance in the whole range from 400–750 nm tended to lie alongside each other, indicating that the concentrations of WP were unlikely to affect the absorbance. In addition, the scattering distributions of absorbance in 500–750 nm were smaller than those in 400 and 450 nm, indicating that the concentrations of WP could affect the absorbances in 400–450 nm, while no significant effect was observed for the 500–750 nm range. As shown above, the influence of both dairy-processing residues on the absorbance in 650–700 nm could be low. Additionally, the absorbance of WPC34 and WP showed the lowest value at 700 nm. Therefore, the influence of dairy-processing residues on absorbance could be reduced at 700 nm of the wavelength for evaluation of the cell growth with OD.

The growth of *C. reinhardtii* was evaluated in modified TAP media containing WPC34 and WP to evaluate the usability of the dairy-processing residues as resources for green alga *C. reinhardtii* (Figure 3, Table 1). *C. reinhardtii* did not grow in the modified TAP medium without WPC34 and WP, simply indicating that *C. reinhardtii* could not use the nitrogen source. On the other hand, *C. reinhardtii* grew in modified TAP medium containing WPC34 or WP because *C. reinhardtii* was able to use WPC34 or WP as nitrogen sources. Cell growth is known to be affected by the least abundant of the required nutrients [45]. The high correlation between the concentration of WPC34 and the maximum OD_700_ indicated that *C. reinhardtii* could use WPC34 as a nitrogen source, which was a depleted source in modified TAP medium. Furthermore, a certain correlation between the doubling time and WPC34 concentration was shown with a coefficient of determination (*R*^2^ = 0.8707). The high correlation between doubling time and WPC34 concentration meant that sufficient amounts of essential nutrients for *C. reinhardtii* growth were simply increased by adding WPC34, resulting in an increment of the maximum OD but no significant change in doubling time, meaning that the nonexistent nutrients were possibly added. 

Next, regarding the low correlation between the concentration of WP and the maximum OD_700_, the low coefficient of determination could be caused by saturation in the range of 10 g∙L^−1^ or higher at a high WP concentration. This suggestion could point out that the high concentration of WP saturated as a necessary nutrient to reach the maximum density of *C. reinhardtii* under the conditions. However, even in the high range of WP concentration, there was no significant correlation between the WP concentration and maximum OD_700_, whereas there was a high correlation between the WP concentration and doubling time. Although the growth rate was enhanced by increasing the WP concentration by providing the necessary amount of essential nutrients for growth like nitrogen sources, the maximum OD_700_ did not improve by increasing the WP concentration, possibly due to the limitation of the maximum cell density under these conditions. A point to keep in mind was that the doubling time in this study was the doubling time of OD_700_ and not the doubling time of simple cell density because the OD value could reflect not only cell density but also cell size in the asexual reproduction cycle. 

The WP and WPC34 prepared in this study contained 81.2 wt% and 54.7 wt% carbohydrates, and 11.9 wt% and 36.4 wt% proteins, respectively (Figure 1). WPC34, treated by the filtration process, has reduced carbohydrate content and enriched protein content compared to WP, which is refined without this process. Despite the total mineral content remaining seemingly unchanged at 2.9 wt% in both WPC34 and WP, the filtration process may have altered the concentration of individual minerals. Given the importance of mineral content in microbial media, the proportion of each mineral in WPC34 and WP was examined (Supplemental Table S2). Minerals such as K, Ca, P, Na, and Mg were present in the order of mg∙g^−1^ in both WP and WPC34, while Fe, Zn, Mo, and Cu were lower, in the order of µg∙g^−1^. The mineral composition of WP, a direct dry product of cheese whey, aligns with previous studies, indicating its general characteristics [46]. Conversely, the high concentrations of K, Ca, P, Na, and Mg in WPC34 suggest that these ions remained bound to proteins and other substances. Whey proteins, which are concentrated in the process of conversion from WP to WPC34, bind specific minerals such as calcium, magnesium, zinc, iron, sodium, and potassium [47]. This could explain the observed higher mineral content in WPC34 compared to WP.

Compared to the culture condition without the mineral addition (Figure 4), the minerals in KCl, K_2_HPO_4_, Na_2_HPO_4_ in WPC34 were sufficient for early growth; however, there was a possibility to improve the final cell density by adding minerals. On the other hand, the amount of KH_2_PO_4_ was sufficient for the initial growth; there was no possibility to improve the maximum cell density by adding this mineral and a negative effect on growth could be shown by further addition of KH_2_PO_4_. Regarding WPC34-containing medium, in the case of K_2_HPO_4_, the concentration of K_2_HPO_4_ was 447.3 mg∙L^−1^, resulting in K^+^ and HPO_4_^2−^ being 200.8 mg∙L^−1^ and 246.5 mg∙L^−1^; in the case of KH_2_PO_4_, the concentration of KH_2_PO_4_ was 698.9 mg∙L^−1^, resulted in K^+^ and HPO_4_^2−^ being 200.8 mg∙L^−1^ and 492.9 mg∙L^−1^ (phosphate ion is normally HPO_4_^2−^ in the pH range of 7–8). The fact that the maximum OD_700_ in the presence of K_2_HPO_4_ was increased and that after adding KH_2_PO_4_ was decreased indicated the threshold level of adding a phosphate source for the growth, showing the possibility of inhibition of the growth with a high concentration of a phosphate source. In addition, the pH could display few effects on the growth since the shifts of pH were within 7 to 8 under all culture conditions with minerals added up to 96 h.

For the lipid contents of the cells cultured in WPC34 and WP-containing media, no significant differences were observed (Figure 5). Normally, *C. reinhardtii* is known to increase the ratio of lipids in the cells under stresses such as nitrogen- and phosphorus-depletion, and a previous study showed that the *C. reinhardtii* strain used in this study increased to 12.1 ± 1.1 wt% of the lipid content ratio under nitrogen-depletion [48]. In comparison, the lipid content in this study was lower than that in the previous report even though the same species was used, so the stress like nitrogen-depletion was weak in both main culture environments containing WPC34 and WP. However, in the cells grown in the WPC34-containing medium, the tendencies of the composition ratios of an increased fatty acid-C14 and a decreased sum of fatty acid-C16 and -C18 suggested that the reaction of fatty acid elongation in the WP-containing medium might be activated but not in the WPC34-containing medium. Li et al. reported that the ratio of the lengths of fatty acids in *C. reinhardtii* cells is shortened under stress such as nitrogen depression [49], possibly indicating that the cells in WPC34-containing medium felt stress but not those in WP-containing medium. In addition, *C. reinhardtii* increases the unsaturation degree of fatty acids under stresses such as salt addition [50], so the cells in WPC34-containing medium felt fewer stresses than those in WP-containing medium.

Cells sense changes in their environment and in response to external stimuli, and then the cells change their metabolism and gene expression. Generally, external stimuli affect the transcription levels of structural genes, and then the levels influence protein expression [51]. Afterwards, protein expression responds to external stimuli by controlling catabolism and anabolism by adjusting the flux and pool of metabolites in cases of functioning as enzymes [52] and/or by controlling the intracellular environment as membrane channels and buffers [53]. In evaluating the cellular control in response to external stimuli, the evaluation of gene transcription levels is more fundamental than the evaluation of protein expression levels or metabolite pools, and that is easier to indicate the cell’s response to stimuli. Therefore, comprehensive gene transcription analysis is a suitable tool to evaluate cellular responses. In this study, the cellular responses in the media containing WPC34 and WP as dairy-processing residue were evaluated by analyzing gene transcription levels comprehensively in the important metabolic pathways such as glycolysis, TCA cycle, and lipid synthesis/decomposition system of *C. reinhardtii* (Figure 6).

Glycolysis is a basic pathway to understand the cellular responses. Especially in *C. reinhardtii*, the pathway is important to evaluate whether the species attempts to switch assimilation to saccharide and dissimilation from saccharide as the cellular response [54]. Therefore, in this study, the glycolytic gene transcription levels in *C. reinhardtii* were evaluated when dairy-processing residue was used as a medium component (Figure 6a). In terms of glycolysis, most of the gene transcription levels in the WP-containing medium were increased compared to those in other medium conditions, indicating the possibility that the cells attempted to activate glycolysis overall by using glucose obtained through hydrolysis of lactose in WP, which normally contains more than 70% lactose as abundant carbohydrate sources [39] compared to other media. Especially, the transcription level of *GAPDH*, which divides the glycolytic system into the phases of the preparatory and pay-off and is a ubiquitous enzyme that reversibly catalyzes the dephosphorylation of 1,3-bisphosphoglycerate to produce glyceraldehyde 3-phosphate and inorganic phosphate [55,56,57], was significantly higher in the WP-containing medium than that in other media, indicating that *C. reinhardtii* cells could activate glycolytic metabolism. The transcription level of *GAPDH* seems to be enhanced when *C. reinhardtii* activates sugar metabolism in glycolysis. *FBP* converts d-fructose 1,6-bisphosphate to d-fructose 6-phosphate, and *PFK*① and *PFK*② convert d-fructose 6-phosphate to d-fructose 1,6-bisphosphate. They are the enzymes that control metabolic reactions in one direction so that these enzymes can control the metabolic flow of sugar assimilation and dissimilation [58]. 

In this study, the transcription levels of *FBP*, *PFK*①, and *PFK*② in the WP-containing medium were significantly higher than those in other media, and the results also indicated that the cells could activate the metabolic flows of both assimilation and dissimilation as sugar metabolism activation. Green algae are known to increase the gene transcript levels of FBP and PFK when sugar metabolism is activated [59], and this fact could reinforce the possibility that the enhancement in transcription levels of *FPB* and *PFK* meant activation of sugar metabolism in this study. *GPMI* and *ENO* are also important enzyme genes in glycolysis as the unique structural enzyme genes controlling the metabolic flow [60], and the fact that the transcription levels of those enzyme genes were improved in the WP-containing medium compared to other media could support the idea of activating glycolysis by adding WP. In particular, *ENO* is an important enzyme acting as a control factor at the crossroads of metabolism from glycolysis to pyruvate [61], and energy metabolism is influenced by the enzymatic activity of ENO [62]. Therefore, the activation of the gene transcription level of ENO in the WP-containing medium might mean the enhancement of energy metabolism. 

Despite being a unique enzyme, the transcription level of only *PGK* in the WP-containing medium did not significantly increase compared to that in other media. 3-phospho-d-glycerate is a substrate related to PGK and it is deeply involved in the synthesis of Ser and the subsequent synthesis of Cys and Gly in plants [63], and the amount of 3-phospho-d-glycerate greatly influences the synthesis of Ser, Cys, and Gly. Therefore, the fact that the transcription level of *PGK* did not increase significantly by adding WP might indicate that the metabolism related to those amino acids was not particularly activated in the WP-containing medium. Glycolysis is an important sugar-metabolic pathway, deeply related to the TCA cycle, lipid production pathway, pentose phosphate pathway involved in ribose synthesis, and the amino acid synthesis pathway, such as Ala, Leu, and Val in photosynthetic organisms [64]. Therefore, as described above, activation of the gene transcription levels in glycolysis in the WP-containing medium might also indicate activation of those pathways.

In *C. reinhardtii*, the TCA cycle is deeply involved in energy production for the preparation for oxidative phosphorylation [54], and is also related to amino acid synthesis containing 2-oxoglutarate and oxaloacetate, which are intermediate metabolites required for the biosynthesis of Glu and Asp, respectively [65,66]. Therefore, the gene transcription levels of the TCA cycle were evaluated to understand the cellular response of *C. reinhardtii* in media containing the dairy-processing residues (Figure 6b). As an overall trend in the TCA cycle, the gene transcription levels in the WP-containing medium were higher than those in other media. Activation of energy production leads to the activation of growth, and the activation of gene transcription levels, especially in the TCA cycle in *C. reinahrdtii*, could correspond to activation of growth through energy production. In fact, as shown in Figure 3, the growth activity, shown as the values of maximum OD_700_ and *µ*_max_ in the WP-containing medium, was higher than in the other media, indicating that the phenomena of the overall activated transcription levels in the TCA cycle in the WP-containing medium would be related to the improvement in the growth activities. In particular, regarding the tendency for improving the gene transcription level in the WP-containing medium, the enhancement of the transcription levels of *PC*, *ACO*, *IDH*1, *OGDH*, and *LSC*1, which are deeply involved in metabolic control as the unique enzymes and *IDH*3①/*IDH*3②, *SDH*1/*SDH*2/*SDH*3, *MDH*1/*MDH*2①/*MDH*2②/*MDH*2③, which as isozymes showed the same trend, as not unique enzymes, strongly suggests activation of the TCA cycle in the WP-containing medium, reinforcing the possibility that this activation leads to improved cell growth. Additionally, in the TCA cycle, the gene transcription levels of the WPC34-containing medium might be improved compared to TAP medium, such as for *DLST*① and *DLST*②, although not as much as the WP-containing medium; the gene transcription levels in TAP medium did not significantly exceed those in the media containing dairy-processing residues except for *DLD*①. Therefore, adding dairy-processing residues could increase the gene transcription levels more than adding NH_4_Cl, indicating that these activations possibly improve metabolism in the TCA cycle.

In *C. reinhardtii*, synthesis and decomposition of lipids implies the assimilation and dissimilation of carbon sources, so understanding the trends of the increment and the decrement of the gene transcription levels in those pathways could help explain how *C. reinhardtii* cells attempt to drive their metabolism energetically. Thus, the gene transcription levels related to the lipid synthesis pathway were firstly evaluated (Figure 6c). In the WP-containing medium, the gene transcription levels except DPP1 in the lipid synthesis pathway showed few tendencies to be prominent as observed in the glycolysis and TCA cycle, so that the lipid synthesis pathway could not be activated as were the glycolysis and TCA cycle. In fact, in *Chlamydomonas* sp., lipid storage is inactive when the cell growth is activated [31], and therefore the results of the transcription levels in this study implied inactivation. Exceptionally, the transcription level of *DPP*1 in media containing dairy-processing residues was higher than that in TAP medium. DPP1 relates lipid production with the reaction catalyzing the dephosphorylation of phosphatidic acid to form diacylglycerols and inorganic orthophosphates [67], so its upregulation could promote lipid production in this study. Malonyl-CoA is deeply involved in the elongation reaction of fatty acids through the action of 3-ketoacyl-ACP synthase [68]. The transcription levels of *ACCA*, *ACCB*, and *ACCC* in the WP-containing medium were higher than those in the WPC34-containing medium. Therefore, as shown in Figure 5, the chain length of fatty acids in the WP-containing medium might have a tendency toward higher contents of fatty acid-C16 and -C18 than that of fatty acid-C14 compared to those in the WPC34-containing medium. Although the transcription level of *ACCD* in the WP-containing medium was lower than that in the WPC34-containing medium, the gene transcription level was the lowest among the *ACCs*, so the effect on the metabolic flow might be low. The enzymes of PLSC and DGAT2 relate the synthesis of lipids by connecting Acyl-CoA to sn-glycerol 3-phosphate [69]. Although these gene transcription levels in the media containing dairy-processing residues tended to be higher than those in TAP medium, lipid production was not activated (Figure 5). In this case, this fact might not significantly affect lipid production.

The gene transcription levels related to the lipid decomposition pathway were secondly evaluated (Figure 6d). The gene transcription levels of MFP2, which is involved in the control of the conversion reaction of trans-hexadec-2-enoyl-CoA and 3-oxohexadecanoyl-CoA, and ACAA1, which mediates the decomposition of acetyl CoA, in the media containing dairy-processing residues were higher than those in TAP medium. This could mean that the activation of lipid decomposition occurred, possibly indicating low lipid contents in the cells grown in dairy-processing residues. These low levels, including ACAA1, which is particularly a regulator of β-oxidation [70], might relate to their low lipid content. Although the gene transcription levels of ACSL, which is a necessary enzyme for reacting fatty acids with CoA, and ACOX1 and ACADM, which are enzymes related to fatty acid desaturation in β-oxidation, in the WP-containing medium were higher than those in the WPC34-containing medium, the transcription levels of MFP2 and ACAA1 as rate-limiting downstream enzymes for lipid decomposition were depressed. Thus, these results could indicate that there was no difference in the lipid content in the cells between the two media using dairy-processing residue.

## 5. Conclusions

In this study, the growth of *C. reinhardtii* and its cellular responses in media containing dairy-processing residues WPC34 and WP as nutritional sources were evaluated. After validating the growth evaluation of *C. reinhardtii* in the media containing the dairy-processing residues, this study revealed that *C. reinhardtii* were able to use WPC34 and WP as nitrogen sources and that the cells could show higher growth in WP-containing medium than that in WPC34-containing medium, explained by the higher specific growth rate and maximum OD_700_. As a result of analyzing the time course profiling of OD_700_ in the WPC34-containing media supplemented with the components of K, P, and Na, the maximum OD_700_ was significantly improved by adding KCl and K_2_HPO_4_, meaning that the material productivity of this strain was possibly increased simply by adding these salts. In the case of *C. reinhardtii*, stresses tend to affect the content and the composition of lipids in the cells. The effects derived from adding WPC34 and WP could not influence the lipid contents in the cells with significant differences and those values were lower than those in normal TAP medium; however, their addition caused ratio differences of fatty acids. Evaluating comprehensively the gene transcription levels in cells, the gene transcription levels in the WP-containing medium tended to be particularly high in the glycolytic system and TCA cycle; however, not in the lipid production system. This result could mean that lipid production was not activated due to vigorous cell growth and active metabolism. Furthermore, the higher transcription level of *ACAA*1 controlling β-oxidation in media containing dairy-processing residues than that in normal TAP medium could be related to the low lipid-contents in the cells. In addition, the gene transcription levels of ACCA, ACCB, and ACCC in the WPC34-containing medium were higher than those in the WP-containing medium, possibly indicating the fatty acid chain lengths. As mentioned above, this study evaluated the use of dairy-processing residues as nutrients by green alga *C. reinhardtii* and the cell responses in the residue-containing medium and the results are meaningful in terms of expanding the use of dairy-processing residues in the future.

## Figures and Tables

**Table 1:** Growth properties of *C. reinhardtii* in media containing WPC34 or WP as nutrients.

Resource	Concentration (g·L^−1^)	Time Range (h)			Maximum Specific Growth Rate (*µ*_max_, h^−1^)			Doubling Time (h)			Maximum OD_700_			Corresponding Figure
-	0	Not detected			Not calculated			Not calculated			Not detected			Figure 3a
WPC34	1	48	~	72	0.009	±	0.001	74.80	±	7.40	0.05	±	0.00	Figure 3a
	3	72	~	96	0.012	±	0.002	57.68	±	14.48	0.12	±	0.00	Figure 3a
	6	24	~	48	0.065	±	0.011	10.77	±	1.73	0.28	±	0.01	Figure 3a
	10	24	~	48	0.144	±	0.072	5.46	±	2.70	0.43	±	0.03	Figure 3a
WP	5	48	~	72	0.019	±	0.000	35.28	±	1.51	0.33	±	0.00	Figure 3d
	10	48	~	72	0.026	±	0.002	26.67	±	1.64	0.63	±	0.03	Figure 3d
	20	72	~	96	0.038	±	0.009	22.74	±	4.13	0.68	±	0.05	Figure 3d
	30	48	~	72	0.058	±	0.004	11.93	±	0.89	0.71	±	0.01	Figure 3d

The data were obtained by 3–8 time-replicate experiments (n = 3–8). Statistically analytic results are shown as SD.

## Data Availability

Data are contained within the article and Supplementary Materials.

## Supplementary Materials

The following supporting information can be downloaded at: https://www.mdpi.com/article/10.3390/microorganisms12040715/s1, Table S1: Primer pairs to perform qPCR in this study, Table S2: Compositions of minerals in WP and WPC34 and maintained ratios of minerals in WPC34 after filtration towards WP, Figure S1: Analyses of scattered Ct values of each candidate housekeeping gene in qPCR.
