# Supplementary material for: Evaluation of Cellular Responses by Chlamydomonas reinhardtii in Media Containing Dairy-Processing Residues Derived from Cheese as Nutrients by Analyzing Cell Growth Activity and Comprehensive Gene Transcription Levels
Source: Microorganisms. 2024 Mar 31;12(4):715. doi: 10.3390/microorganisms12040715 (PMC11052199; doi:10.3390/microorganisms12040715)
Supplement: Supplementary file 1 [file microorganisms-12-00715-s001.zip › microorganisms-2930842-supplementary.pdf]

Supplemental Data

Supplemental Table S1

Primer pairs to perform qPCR in this study.

Gene name (NCBI-GeneID)	Abbreviation	Forward/ Reverse	Sequence alignment
ATPase beta chain (2717034)	*ATPS*	F	gaccctgctcctgctacaac
		R	ggtcaaccgcagggtaaata
actin-related protein Arp2/3 complex, subunit Arp2 (5717667)	*Arp2*	F	agcaccagcaactgaaggac
		R	atctcctcccatgactgcac
actin-related protein Arp2/3 complex, subunit Arp3 (5721474)	*Arp3*	F	cgggagcagtaccaggagta
		R	tgcgtctcggaagacgtagt
actin related protein Arp2/3 complex, subunit ARPC4 (5722039)	*Arpc4*	F	ttagggataacccggagctt
		R	gctgacgcgtacagagttga
glucose-6-phosphate isomerase (5728866)	*GPI*	F	caacaacatcgacgagcact
		R	aggaaggacacgttccacag
fructose-1,6-bisphosphatase I (5716571)	*FBP*	F	gtggtgtccaacgaggtctt
		R	agttgcccgagtaggtctcc
6-phosphofructokinase 1 (5722005)	*PFK*①	F	gacagcccctttattgtgga
		R	gctctcaaactccaccgtgt
6-phosphofructokinase 1 (5719698)	*PFK*②	F	gcctggtgaacaagctgact
		R	aagcccctgaatccgtactt
fructose-bisphosphate aldolase, class I (5725868)	*ALDO*①	F	gtgtggagaacaccgaggac
		R	cagcgtctcctcaaacatga
fructose-bisphosphate aldolase, class I (5725430)	*ALDO*②	F	tatggaagacacggggaatg
		R	tgctaggatgcccttctcag
fructose-bisphosphate aldolase, class I (5726208)	*ALDO*③	F	tgaaggagcagaacatcgtg
		R	ccggccttgtagtactcagc
fructose-bisphosphate aldolase, class I (5727515)	*ALDO*④	F	cgcaaatagctgcacaactc
		R	caagcgcttcccaatagttc
glyceraldehyde 3-phosphate dehydrogenase (phosphorylating) (5728718)	*GAPDH*	F	atgactcggtccacaagacc
		R	ctcgggtcgctctcagtaaa
phosphoglycerate kinase (5725034)	*PGK*	F	ggtggcatgatcttcacctt
		R	catctcctccagcttcttgg
2,3-bisphosphoglycerate-independent phosphoglycerate mutase (5721822)	*GPMI*	F	aagcaggagcagtacctgga
		R	cttgtacttgccgctcttga
enolase (5728533)	*ENO*	F	gcctcgtcggagttctacac
		R	gcatctgctccttggtcttc
pyruvate carboxylase (5722077)	*PC*	F	cgaggagtggtccaacatct
		R	gggtcaccttgacgatgtct
citrate synthase (5728523)	*CS*①	F	agtacatcccgccctcctac
		R	ctggtacatgcgcatcatct
		F	attggctcggttgagaacat

citrate synthase (5721175)	*CS*②	R	tcgaagttgcgatacactcg
ATP citrate (pro-S)-lyase (5727439)	*ACLY*①	F	ttcttcggcaagaacgagat
		R	ggaaggaggcgtagttgatg
ATP citrate (pro-S)-lyase (5726448)	*ACLY*②	F	tatgacgctaagcggcttct
		R	aggcatttgtgtttgcagtg
aconitate hydratase (5715601)	*ACO*	F	tgtgctgatcaaggtgaagg
		R	tgagcaggttgttggagatg
isocitrate dehydrogenase (5724231)	*IDH1*	F	cctggacctgaagatcgtgt
		R	cacattgtgctccttgatgg
isocitrate dehydrogenase (NAD+) (5720461)	*IDH3*①	F	caaggagctggacctttacg
		R	ggacggtgaccaggttaatg
isocitrate dehydrogenase (NAD+) (5722833)	*IDH3*②	F	agaacacaaacacgcagtcg
		R	ctctccctccgtgttctcac
2-oxoglutarate dehydrogenase E1 component (5718469)	*OGDH*	F	ccacatcggctacgagtaca
		R	ttctgctgcttggtgaactg
dihydrolipoyl dehydrogenase (5715128)	*DLD*①	F	gaccgtgttcacctctgacc
		R	tgtacacgtcgctgaactcc
dihydrolipoyl dehydrogenase (5720812)	*DLD*②	F	agttcaagatgggcaccaag
		R	acgtcgcactccatcttctc
2-oxoglutarate dehydrogenase E2 component (dihydrolipoamide succinyltransferase) (66054139)	*DLST*①	F	tcttttcctccacctcctca
		R	ttgacgttgtgcttctccag
2-oxoglutarate dehydrogenase E2 component (dihydrolipoamide succinyltransferase) (5718007)	*DLST*②	F	tggagaagcacaacgtcaag
		R	atctcatcgccctcaatcac
succinyl-CoA synthetase alpha subunit (5718710)	*LSC1*	F	tggcgtcttttccttactgc
		R	cgcaagtgcagaagactcag
succinate dehydrogenase (ubiquinone) flavoprotein subunit (5715870)	*SDH1*	F	agatcatgaagcccaacacc
		R	tgttctgcatcaccttctgc
succinate dehydrogenase (ubiquinone) iron-sulfur subunit (5721979)	*SDH2*	F	ctactggtggaacagcgaca
		R	gcagcggtagagcttgtagg
succinate dehydrogenase (ubiquinone) cytochrome b560 subunit (5715192)	*SDH3*	F	tcgtacgccatcatctacca
		R	aggaccttgctgctcatctc
fumarate hydratase, class I (5722144)	*FUMA*	F	agtacgccaaggaccacatc
		R	actcgtccacgtagctgtcc
fumarate hydratase, class II (5715189)	*FUMC*	F	aattcgcaccgttttattgc
		R	tctggttatggtggtgtgga
malate dehydrogenase (5720512)	*MDH1*	F	tgtactcctaccccgtcacc
		R	gtcgatgggtagaccctgaa
malate dehydrogenase (5718730)	*MDH2*①	F	ctttgtgtcggaggctaagg
		R	gtcatggccttcttctcagc
malate dehydrogenase (5728232)	*MDH2*②	F	cactgaaccgcattcaaaag
		R	acacgtaggggctcatcttg
malate dehydrogenase (5728707)	*MDH2*③	F	ctacttcgcctccaaggtca

		R	gccttctcgtagtccgacag
acetyl-CoA carboxylase carboxyl transferase subunit alpha (5722616)	*ACCA*	F	gaacagaagcatgccaaaca
		R	ccgttgcgtcgtctgtagta
acetyl-CoA carboxylase biotin carboxyl carrier protein (5715506)	*ACCB*	F	ttgtcgggtttgtacgtttg
		R	ctttgtagccaagccctctg
acetyl-CoA carboxylase, biotin carboxylase subunit (5728708)	*ACCC*	F	tcttttcgctcatgcagatg
		R	aggtgaggatggcgatgtag
acetyl-CoA carboxylase carboxyl transferase subunit beta (5727859)	*ACCD*	F	tcctccgagtcctacctcaa
		R	gctcagacaggaagccgtag
1-acyl-sn-glycerol-3-phosphate acyltransferase (5720374)	*PLSC*	F	aactcagactccgctcctca
		R	ccatcatcgtcacaaacagc
diacylglycerol diphosphate phosphatase /phosphatidate phosphatase (5727801)	*DPP1*	F	cgtgcttgactactggcact
		R	gggtagatgaggcggtacac
diacylglycerol O-acyltransferase 2 (5718859)	*DGAT2*	F	cctctttcttagggccgaac
		R	gtgtagatggcgctgacgta
long-chain acyl-CoA synthetase (5728486)	*ACSL*①	F	gcccctctgattcctacctc
		R	atgtcaccctgccagtatcc
long-chain acyl-CoA synthetase (5716991)	*ACSL*②	F	agcgcttggtagaggctaca
		R	agcttgtcgccgtaagtgac
long-chain acyl-CoA synthetase (5716500)	*ACSL*③	F	tacacgtggatgacctacgc
		R	agccactccttgcagttgac
long-chain acyl-CoA synthetase (5719061)	*ACSL*④	F	caaggaggacaagctcaagg
		R	aggtcgtcctccacagagaa
acyl-CoA oxidase (5727823)	*ACOX1*①	F	aggcgaaggtgtaccagatg
		R	cctgcaggattaccttgagc
acyl-CoA oxidase (5725038)	*ACOX1*②	F	ctacgttccgcaatacgaca
		R	agtccgtcaccgaaatgaag
acyl-CoA oxidase (5717664)	*ACOX1*③	F	gacctacgcctaccacttcg
		R	ggatagcacgtggatggtct
acyl-CoA oxidase (5724745)	*ACOX1*④	F	tgattctgaagacgcacacc
		R	gctcctttccaccattcaga
acyl-CoA oxidase (5724917)	*ACOX1*⑤	F	caccagcaagatccagaaca
		R	cactggctgaactccatgtc
acyl-CoA dehydrogenase (5721471)	*ACADM*	F	actaccgcctcgacaacatc
		R	gtagggcaggcagttgtagg
enoyl-CoA hydratase /3-hydroxyacyl-CoA dehydrogenase (5724874)	*MFP2*	F	ttgacatcagccagttccag
		R	catcgttgatggagttgtcg
acetyl-CoA acyltransferase 1 (5722736)	*ACAA1*	F	agatcaccatcagcgaggac
		R	tgccgttcttcttgaacaca
acetyl-CoA C-acetyltransferase (5720517)	*ACAT*	F	gcttcgaaagcatgagcaac
		R	tcccatagtccgtccttcac

Supplemental Table S2

Compositions of minerals in WP and WPC34 and maintained ratios of minerals in WPC34 after filtration towards WP

The maintained ratios (%) were obtained by calculating with formula: 100 × (mineral concentration in WPC34/(3.1 × mineral concentration in WP)) and the value of 3.1 in the formula as a factor of the protein ratio compared to WP described in *3.1. Compositions of Powders of WPC34 and WP*.

	Mineral concentration ($mg \cdot g^{-1}$)					Mineral concentration ($\mu g \cdot g^{-1}$)					
	K	Ca	P	Na	Mg	Fe	Zn	Mo	Cu	Mn	Co
WP	13.4	5.46	5.81	5.21	0.98	1.3	1.2	0.53	0.1	Not detected	Not detected
WPC34	11.2	7.08	5.75	3.05	1.05	3.6	3.4	0.80	0.3	Not detected	Not detected
Maintained ratio (%)	27.9	43.2	33.0	19.5	35.7	92.3	94.4	50.3	100.0	Not calculated	Not calculated

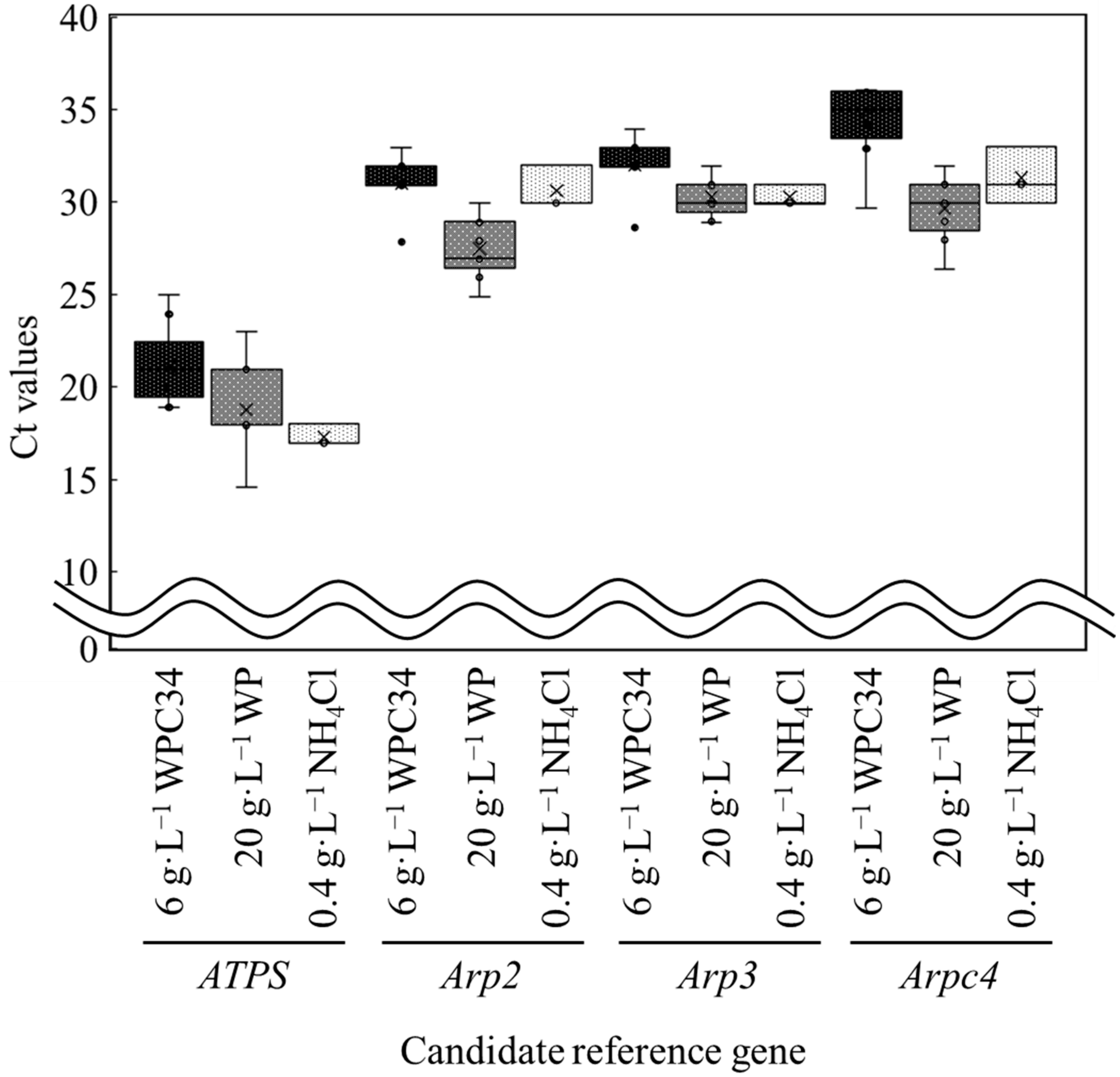

Supplemental Figure S1 Analyses of scattered Ct values of each candidate housekeeping gene in qPCR

Ct values of candidate reference genes were monitored in performing qPCR in modified TAP media containing WPC34 adjusted to 6 g·L^{-1} (WPC34-containing medium), WP adjusted to 20 g·L^{-1} (WP-containing medium), NH_4Cl adjusted to 6 g·L^{-1} (TAP medium). Error bars indicate SD supported by six replicate experiments (n = 6) in media containing dairy-processing residues and three replicate experiments (n = 3) in TAP medium. Reference genes were *ATPS*: *ATPase beta chain of ATP synthase*, *Arp2*: *actin-related protein Arp2/3 complex*, *subunit ARP2*, *Arp3*: *actin-related protein Arp2/3 complex*, *subunit Arp3*, and *Arpc4*: *actin-related protein Arp2/3 complex, subunit ARPC4*.
